# Natural Products: Bioactivity, Biochemistry, and Biological Effects in Cancer and Disease Therapy

**DOI:** 10.1155/2013/713480

**Published:** 2013-08-22

**Authors:** Hsueh-Wei Chang, Li-Yeh Chuang, Sanjay Guleria, Sammia Yasmin

**Affiliations:** ^1^Department of Biomedical Science and Environmental Biology, Cancer Center, Kaohsiung Medical University Hospital, Kaohsiung Medical University, Kaohsiung 80708, Taiwan; ^2^Department of Chemical Engineering & Institute of Biotechnology and Chemical Engineering, I-Shou University, Kaohsiung 84001, Taiwan; ^3^Division of Biochemistry and Plant Physiology, SK University of Agricultural Sciences and Technology, Jammu, Chatha 180009, India; ^4^School of Science & Technology, University of Management & Technology, Lahore 54000, Pakistan

The drug discovery for chemoprevention and chemotherapy remains a challenge. Natural products-derived extracts and compounds are frequently reported to discover therapeutic agents for disease and cancer.

The overall scenario of this special issue of The Scientific World Journal presents the recent advances in biological function of selected natural products for cancer and disease therapy in terms of crude extracts and components. Some studies describe the bioinformatics tool to help to investigate the field of natural products.

The papers by S. Guleria et al. and C.-C. Lee et al. provide the essential oil and/or extracts of herb *Zanthoxylum alatum* and *Zingiber officinale* for its antioxidant and antimicrobial properties, respectively. O. O. Igbinosa et al. and C.-C. Lee et al. provide the animal experiments using extractsfrom* Jatropha curcas* (Linn) leaf and from supercritical carbon dioxide extracted ginger, respectively. Three studies (F. M. Al-Jasass and M. S. Al-Jasser, X.-W. Chen et al., and C.-Y. Lo et al.) focus on biological functions of the compounds from Saudi Arabia herbs, Chinese herb Huang Lian (*Rhizoma coptidis*), and from *Alpinia galangal*, respectively. Further, C.-Y. Yen et al. provide the toxicological study for cardiotoxin III in growth inhibition of oral cancer. C.-Y. Lin et al. provide a review article for the chemoprevention of cytochrome P450 in oral potentially malignant disorders (OPMDs) patients in terms of betel quid metabolism. S.-S. Liang et al. introduce the novel technique for online monitoring oxidative products and metabolites of nicotine based on tandem mass spectrometry.

Some papers introduce the bioinformatics methods or resources to study or review the natural products-related studies. L. Wang et al. introduce the gene ontology (GO) network for the systems-theoretical analysis of human hepatocellular carcinoma. Y.-C. Lin et al. provide the database (TIPdb) for anticancer, antiplatelet, and antituberculosis phytochemicals from indigenous plants in Taiwan. Three papers (W.-H. Huang et al. and J.-Y. Tang et al.) provide the drug discovery for cancer and disease therapy in terms of RNA editing, alternative splicing, and long noncoding RNAs as well as the summary for their bioinformatics resources.

*Hsueh-Wei Chang* *Hsueh-Wei Chang* *Li-Yeh Chuang* *Li-Yeh Chuang* *Sanjay Guleria* *Sanjay Guleria* *Sammia Yasmin* *Sammia Yasmin*

